# Non-stationary time-varying vehicular channel characteristics for different roadside scattering environments

**DOI:** 10.1038/s41598-022-18592-z

**Published:** 2022-08-22

**Authors:** Changzhen Li, Wei Chen, Zhonghui Pei, Fuxing Chang, Junyi Yu, Fan Luo

**Affiliations:** 1 School of Information Engineering, Wuhan University of Technology, Wuhan, 430070 China; 2 School of Automation, Wuhan University of Technology, Wuhan, 430070 China; 3 Beijing MetaRadio Technologies Co., Ltd, Beijing, 100028 China

**Keywords:** Electrical and electronic engineering, Information technology

## Abstract

With the deep integration of wireless communication technology and automobile industry, vehicular communication has become one of the key technologies supporting the development of Internet-of-vehicle. Due to the high-speed mobility of vehicles and the rapid change of the propagation environments, vehicle-to-vehicle (V2V) wireless communication channels are generally non-stationary. Meanwhile, the variability of V2V channel characteristics is obvious in different scattering environments. Focusing on these research points, this paper presents the analysis and comparison of V2V channel characteristics for different scattering scenarios based on a series of 5.9 GHz channel measurements. The measurement data are collected from the iron bridge, the soundproof wall, and the road lamp scenarios. The stationary time and frequency are investigated on the basis of method of local scattering functions. The classical channel characteristics, including power delay profile, Ricean K-factor, root means square (RMS) delay spread and RMS Doppler spread are extracted following the propagation principle. Furthermore, considering the source and birth-death process of multi-path components (MPCs) in different scattering propagation environments, cluster identification and statistical results are presented and compared. The different values of the channel parameters and the different performance of the channel under different scattering environments can help us understand the V2V channel deeply. The research results can be used for the design and optimization of vehicular communication systems in different scattering environments.

## Introduction

Internet-of-vehicle (IoV) is a significant technology to achieve the goals of intelligent vehicles and intelligent transportation systems. It is one of the most important application scenarios of the fifth generation mobile communication technology and a popular research direction in the current Internet of things industry^1,2^. As one of the important basic safeguard technologies, vehicle-to-vehicle (V2V) wireless communication is an essential part of the IoV technology, which can supply a low-delay, high-speed, and secure data transmission service.

Wireless communication between vehicles generally occurs in complicated propagation environments. The communication performance and channel characteristics, to a large extent, are affected by surroundings, especially in the propagation scenarios with abundant scatterers. Compared with the traditional cellular network, V2V communication systems have many different characteristics, such as the low heights and high-speed mobility of both transmitter and receiver antennas^3^. Therefore, the influence on the V2V channel from surrounding scatterers is obvious and non-ignorable. Because it can result in the non-stationary performance of V2V channels^4^.

Indeed, the non-stationary of the channel has attracted more and more attention in the research and analysis of vehicular communications. A lot of channel models focusing on the non-stationary characteristic of V2V channels have been proposed^5–7^. In contrast, there is insufficient literature to study the impact on vehicular communications from different scattering scenarios, such as scatterers with different sizes and structures.

On the non-stationary characteristic of V2V channels, lots of research efforts focus on the construction of geometry-based channel models. Following the theoretical foundation in the classic textbook^8^, the ellipse model is the most popular one in the channel modeling research. $Jiang\, et.\, al$^9^ built a vehicular channel model, using the ellipse model to depict roadside scattering environments. But vehicles in this reference are assumed to be static and described as a two-circle model. Using the similar method, in the channel modeling of the reference by $Liang\, et.\, al$^10^, the static scatterers on the both roadsides are assumed to be uniformly distributed on time-varying ellipses, and the mobile scatterers are uniformly distributed in time-varying segments of the road. However, the scatterers can also be assumed to be randomly distributed on an ellipse with two moving vehicles being its foci^11^.

For the special propagation scenarios, some different geometrical models are used to conduct the channel modeling work as well. For the tunnel environments, $Jiang\, et.\, al$^12^ propose that a two-cylinder model can be used to describe moving vehicles, as well as a multiple confocal semi-ellipsoid model can be used to depict internal surfaces of tunnel walls. $Zhao\, et.\, al$^13^ proposed a geometry-based stochastic scattering model, in which a three-dimensional two-cylinder and a two-dimensional multi-ring are respectively used to describe the stationary and the moving scatterers.

In addition, other models are also used in the non-stationary V2V channel modeling work. For example, in the model of the reference by $Li\, et.\, al$^14^, considering the effects of different vehicles scattering on V2X channels, the authors treat vehicles as the scattering centers. In the proposed geometrical model for the V2V channel in the reference by $Cheng\, et.\, al$^15^, small scattering objects along the roadside are assumed to be uniform linear distributed.

It can be found from above related work that most existing research have partiality for channel modeling on the scattering propagation environments. However, the differences of the influence between different scatters are rarely involved. Meanwhile, as one of important means for the analysis of wireless communication, channel measurement should also be carried out to extract typical channel characteristics, obtain some reliable and real results, and verify the effectiveness of the proposed models.

In order to describe and characterize the non-stationary vehicular channel for the different scattering scenarios, time-varying characteristics in the areas of iron bridge, soundproof wall, and road lamp are measured and analyzed in this paper. Based on the 5.9 GHz channel measurements, the multi-path components (MPCs) caused by the typical roadside scatterers are presented. The time-varying power delay profile, RMS delay spread and RMS Doppler spread are analyzed. The differences of influence on channel characteristics from different scatterers are compared. The main contributions of this paper are as follows.

- Differences of the contributing MPCs from investigated scatters are analyzed. In this paper, the scattering effects caused by different surrounding environments on both sides of the road are distinguished. Characteristics of MPCs from different scatterers are analyzed.
- The influence on vehicular channel characteristics caused by different scatterers is compared. In this paper, we carried out 3 V2V channel measurement campaigns in different scattering scenarios, including iron bridge, soundproof wall, and road lamp cases. Differences of scattering effect caused by different scatterers are explored.
- Stationary times in the different scattering propagation scenarios are extracted. In this paper, considering the non-stationary of V2V channels, the stationary times in the iron bridge, soundproof wall, and road lamp propagation environments are calculated, respectively.
- Cluster identification and statistical characteristics of MPCs caused by different scatterers are conducted. In this paper, considering the source and birth-death process of MPCs, cluster identification for the power delay profile and statistical analysis of inter-cluster interval and intracluster decay time constant are performed.

The remainder of this paper is organized as follows. Section “Measurement campaign” gives the description of V2V channel measurement campaigns. In Section “Time-varying channel characteristics”, time-varying channel characteristics including stationary time, power and delay of MPCs, RMS delay spread and RMS Doppler spread in different scattering scenarios are analyzed. Section “Cluster identification and statistical results” presents cluster identification and statistical results of MPCs. Finally, Section “Conclusion” draws the conclusions.

## Measurement campaign

### Measurement scenarios

The measurement campaigns are conducted in Wuhan, China. The measurement scenarios are set to three kinds of cases with different roadside scattering environments, including iron bridge, soundproof wall, and road lamp cases.

**Figure 1:**
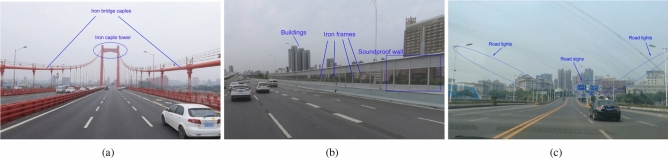
The measurement scenarios. (**a**) Iron bridge. (**b**) Soundproof wall. (**c**) Road lamp.

- **Scenario 1: Iron bridge.** Bridge is a common road structure in some cities with inland rivers or lakes. In this measurement, the bridge is a suspension bridge over the Yangzte river, as shown in Fig. 1a. The main structures of the bridge include three large iron cable towers and several iron cables on both sides of the bridge. In our measurement, the two measurement vehicles drove in the same direction, passing through three iron towers in turn. During the measurement, the TX vehicle firstly keep driving behind the RX vehicle. After about 10 s, the TX vehicle overtook the RX vehicle. Due to the large height of the iron towers, there is almost no other obstruction between vehicles and the top of the tower, which means an obvious LOS path will exist between measurement vehicles and iron towers. However, the iron cables beside the bridge and the other parts of the iron towers will result in reflection effects.
- **Scenario 2: Soundproof wall.** Soundproof wall appears generally on the viaducts built in urban areas, aiming at reducing the noise and guaranteeing the normal and quiet lives of residents near the viaducts. The structure of soundproof wall normally consists of sound barriers and metallic frames, as shown in Fig. 1b. The existence of soundproof wall makes the propagation environment become a semi-enclosed scene, which will cause some different channel characteristics comparing to that in the traditional propagation scenarios. At the same time of this measurement, some other vehicles passed by the measurement vehicles. Meanwhile, there are also many buildings with large heights beside the viaduct. Therefore, the passing vehicles and buildings will result in some reflection paths.
- **Scenario 3: Road lamp.** This scenario is selected on a bridge with a wide view, as shown in Fig. 1c. In this measurement, the vehicles drove between two rows of road lamps neatly distributed on the roadside. The road lamps are propped up by iron poles. Except the distant buildings, the main reflection source will be the road lamps beside the road, the metallic traffic signs, and other passing vehicles in this case.

According to the description above, we can find that what the three measurement scenarios have in common is the presence of reflections caused by scatterers beside the road. However, it should be noted that the scatterers are not the same. Therefore, whether it will result in different influence on the vehicular channel characteristic is the issue we need to explore in the next work.

### Measurement equipment

In this paper, our measurement campaigns are conducted using the radio channel sounder provided by super radio AS and Norwegian University of Science and Technology (NTNU)^16^. The measurement system is composed of the following parts:

- TX and RX: The transmitter (TX) of the channel sounder performs single-input single-output (SISO) measurement and emits a chirp signal. The power of TX part is 16 dBm. The carrier frequency is set to 5.9 GHz with a frequency bandwidth of 100 MHz. The receiver (RX) can receive 1933 chirps per second. Every chirp signal contains 2560 samples.
- TX antenna: TX antennas are installed on the roof of transmitter vehicles at the heights of 1.53 m, 1.57 m and 1.57 m with the antenna gains of 2 dBi, 2 dBi and 2 dBi for measurements 1, 2 and 3, respectively.
- RX antenna: RX antennas are fixed to the roof of receiver vehicles. The heights of them for measurements 1, 2 and 3 are 1.50 m, 1.50 m and 1.78 m, and the antenna gains are 2 dBi, 2 dBi and 10 dBi, respectively.
- Others: During each measurement, two computers are used to collect and save real-time information, including measurement data, global positioning system (GPS) data and speeds of TX and RX vehicles. Moreover, videos are recorded during the entire measurements.

**Table 1:** Measurement Parameters.

	Measurement 1	Measurement 2	Measurement 3
Center frequency $f_c$	5.9 GHz	5.9 GHz	5.9 GHz
Bandwidth *B*	100 MHz	100 MHz	100 MHz
Delay resolution $\Delta \tau _{min}$	10 ns	10 ns	10 ns
Chirp interval $T_C$	517 us	517 us	517 us
TX power $P_{TX}$	16 dBm	16 dBm	16 dBm
TX antenna gain $G_{TX}$	2 dBi	2 dBi	2 dBi
RX antenna gain $G_{RX}$	2 dBi	2 dBi	10 dBi
TX antenna height $h_{TX}$	1.53 m	1.57 m	1.57 m
RX antenna height $h_{RX}$	1.50 m	1.50 m	1.78 m

Tab. 1 gives the detailed parameter settings for the three measurements. All the antennas used in the measurements are omni-directional in order to collect measurement data more accurately.

## Time-varying channel characteristics

### Stationary time

Vehicular communication usually occurs in a rapidly changing and mobile driving environment. In these propagation environments, scatterers are distributed on both sides of the road. With the movement of vehicles, the scatterers will result in the vehicular channel being a non-stationary fading process^17,18^.

For non-stationary channels, stationary time is an important characteristic, in which the analysis of vehicular channel can be simplified under the assumption of wide-sense-stationary and uncorrelated scattering (WSSUS). Therefore, in order to measure the stationary time, the collinearity of the local scattering function is used in this paper. The value of collinearity varies from 0 to 1. A larger value represents a similar power spectral density between the neighboring local scattering functions, where the fading process can be considered quasi-stationary. And the stationary time is defined as the time range where the collinearity exceeds a threshold of $\alpha _{{\mathrm{th}}}$ = 0.9^5,19^.

Based on the methodology in the reference by $Bernad\acute{o}\, et.\, al$^19^, the collinearity in time can be defined as

1 $$\begin{aligned} c_{{\mathrm{t}}}[k_{{\mathrm{t}}},k_{{\mathrm{t}}}+\Delta k_{{\mathrm{t}}}]=\frac{\sum \limits _{n=0}^{N-1}\sum \limits _{p=-M/2}^{M/2-1}\sum \limits _{k_{{\mathrm{f}}}=-N/2}^{N/2-1}L[k_{{\mathrm{t}}},k_{{\mathrm{f}}};n,p]\odot L[k_{{\mathrm{t}}}+\Delta k_{{\mathrm{t}}},k_{{\mathrm{f}}};n,p]}{\Vert L^{(k_{{\mathrm{t}}})}\Vert _2 \cdot \Vert L^{(k_{{\mathrm{t}}}+\Delta k_{{\mathrm{t}}})}\Vert _2} \end{aligned}$$

where $L[k_{{\mathrm{t}}},k_{{\mathrm{f}}};n,p]$ is the estimate of a time-frequency dependent scattering function on the basis of local scattering function^19^. The $\Vert \cdot \Vert _2$ operation on $L^{(k_t)}$ is the vectorized local scattering function at a given time instant $k_t$. $n\in \{0,\cdots ,N-1\}$ denotes the delay index, and $p\in \{-M/2,\cdots ,M/2-1\}$ is the frequency index. Meanwhile, $k_{{\mathrm{t}}}$, $k_{{\mathrm{f}}}$ are the index of the consecutive stationary region in time and frequency.

**Figure 2:**
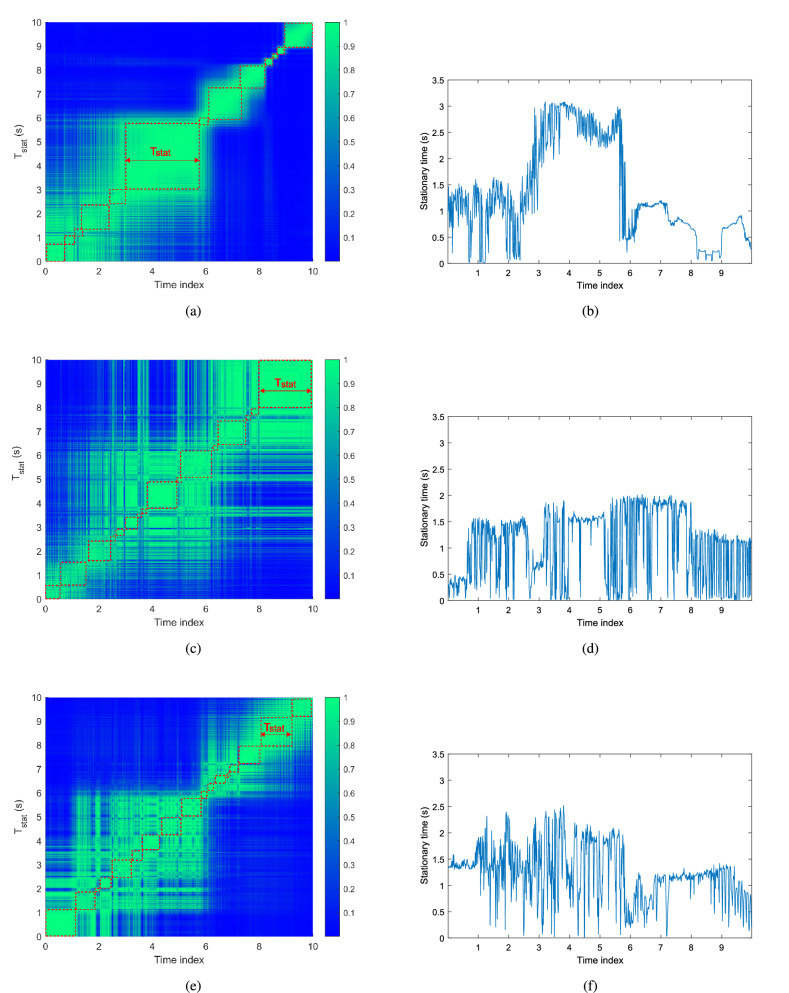
Collinearity in time and stationary time for the measurements. (**a**, **b**) Iron bridge. (**c**, **d**) Soundproof wall. (**e**, **f**) Roadside lamp.

In our analysis, we define $t_{{\mathrm{s}}}$, $M_t$, $\Delta t$ as time resolution, dimension of the minimum stationary region in time, and time shift between consecutive stationary region, respectively. The stationary time $T_{{\mathrm{stat}}}$ can thus be calculated by the Equation (2).

2 $$\begin{aligned} T_{{\mathrm{stat}}}[k_{{\mathrm{t}}}]=t_{{\mathrm{s}}}(M_t-\Delta t)+t_{{\mathrm{s}}}\Delta t\left( \sum \limits _{\Delta k_{{\mathrm{t}}}=1-k_{{\mathrm{t}}}}^{K_{{\mathrm{t}}}-k_{{\mathrm{t}}}}\alpha [k_{{\mathrm{t}}},k_{{\mathrm{t}}}+\Delta k_{{\mathrm{t}}}]\right) \end{aligned}$$

Here, $\alpha$ is the indicator function, which is defined as

3 $$\begin{aligned} \alpha [k_{{\mathrm{t}}},k_{{\mathrm{t}}}+\Delta k_{{\mathrm{t}}}]= {\left\{ \begin{array}{ll} 1&{} c_{{\mathrm{t}}}[k_{{\mathrm{t}}},k_{{\mathrm{t}}}+\Delta k_{{\mathrm{t}}}]>\alpha _{{\mathrm{th}}}\\ 0&{} {\text {otherwise}}. \end{array}\right. } \end{aligned}$$

According to the method in the reference by $Bernad\acute{o}\, et.\, al$^19^, we set dimension of the minimum stationary region to $M_t$ = 40 samples with 20.7 ms. And then, the sliding shift, a half of $M_t$, $\Delta t$ is equal to 20 with a resolution of 10.3 ms in $T_{{\mathrm{stat}}}$. The analysis in frequency domain are conducted in exactly the same way, except a different dimension minimum stationary region $N_f$, where $N_f$ is 512 with 20 MHz and the sliding shift in frequency of $\Delta f$ is 128 with a resolution of 5 MHz in $F_{{\mathrm{stat}}}$.

Fig. 2a–e show the collinearity in time of the measurements in iron bridge, soundproof wall, and roadside lamp propagation environments, respectively. For the collinearity in time, a high value between $k_t$ and $\Delta k_t$ can be considered as a similar power spectral density without rapid change in the channel during this time region. Meanwhile, the stationary time after applying the threshold of $\alpha _{{\mathrm{th}}}$ = 0.9 is shown in Fig. 2b–f. Furthermore, the minimum $T_{{\mathrm{stat}}}$ and $F_{{\mathrm{stat}}}$, the 5% outage probability, mean and standard deviation values of $T_{{\mathrm{stat}}}$ and $F_{{\mathrm{stat}}}$ are presented in Tab. 2.

It can be found from the above results that the values of minimum stationary time obtained from the three measurements are similar (around 10.35 ms). However, values of the 5% outage probability and average stationary times are different. The statistical results indicate that, for the stationary time, the average values and values of the 5% outage probability are larger in the iron bridge and road lamp scenarios than the values obtained from the soundproof wall scenario. The statistical results of stationary frequency are similar to the results of stationary time. It means that the V2V channels in the iron bridge and road lamp scenarios are more stationary than that in the soundproof wall scenario. The reason is that the propagation environments are relatively open in the iron bridge and road lamp scenarios with stable iron chains and metal poles. In contrast, the existence of soundproof walls on both sides of the road forms a relatively closed propagation environment, which leads to the surrounding vehicles with great mobility and randomness being the main scatterers. Therefore, these factors result in a non-stationary channel in the soundproof wall scenario. Our result is consistent with the conclusion drawn from the in-tunnel and on-bridge scenarios in the reference by $Bernad\acute{o}\, et.\, al$^19^.

**Table 2:** Statistical results of the stationary time.

Scenarios	$T_{{\mathrm{stat}}}$				$F_{{\mathrm{stat}}}$			
	min	5% out	mean	std	min	5% out	mean	std
Iron Bridge	10.35 ms	0.37 s	3.26 s	2.51 s	5 MHz	20 MHz	66.88 MHz	18.99 MHz
Soundproof Wall	10.35 ms	0.09 s	1.55 s	1.04 s	5 MHz	10 MHz	57.18 MHz	22.59 MHz
Street Lamp	10.35 ms	0.68 s	2.49 s	1.22 s	5 MHz	30 MHz	69.14 MHz	16.74 MHz

### Power delay profile

In the analysis of wireless channel characteristics, power delay profile (PDP) is generally used to describe the received power of MPCs within a period of propagation delay from $\tau$ to ($\tau$ + $\Delta$ $\tau$). After processing the channel measurement data, we can get discretized channel impulse response (CIR) $h(T_{\mathrm {C}} n, \Delta \tau _{\min } m)$ by inverse Fourier transform on the channel transfer function with respect to the frequency. Then, the instantaneous PDP $P(T_{\mathrm {C}} n, \Delta \tau _{\min } m)$ can be obtained by equation (4).

4 $$\begin{aligned} P(T_{\mathrm {C}} n, \Delta \tau _{\min } m) = \left| h(T_{\mathrm {C}} n, \Delta \tau _{\min } m) \right| ^{2} \end{aligned}$$

where, $T_{\mathrm {C}}n$ denotes the measurement time *t*, $n\in \{0, 1, \cdots , N_c-1\}$. $N_c$ is the total number of chirps within a time unit. $\Delta \tau _{\min } m$ represents the delay $\tau$, $m\in \{0, 1, \cdots , N_s-1\}$. $N_s$ is the number of samples per chirp. In this paper, according to the parameter setup of the channel sounder, $N_c$ is 1933 per second and $N_s$ is 2560.

We then define $N_{\mathrm {w}}$ as the length of quasi-stationary window and $j\in \{1, 2,\ldots , N_J\}$ as the window index. $N_{\mathrm {J}}$ represents the number of quasi-stationary windows. $t_j=N_{\mathrm {w}}\cdot (j-1)\cdot T_{\mathrm {C}}$ denotes the time of the *j*-th window. Thus, the average power delay profile (APDP) in the *j*-th window can be given by equation (5).

5 $$\begin{aligned} {\overline{P}} (t_{j}, \Delta \tau _{\min } m) = \frac{1}{N_{\mathrm {w}}} \sum \limits _{n = (j-1)\cdot N_{\mathrm {w}}+1}^{j \cdot N_{\mathrm {w}}} P(T_{\mathrm {C}} n, \Delta \tau _{\min } m) \end{aligned}$$

**Figure 3:**
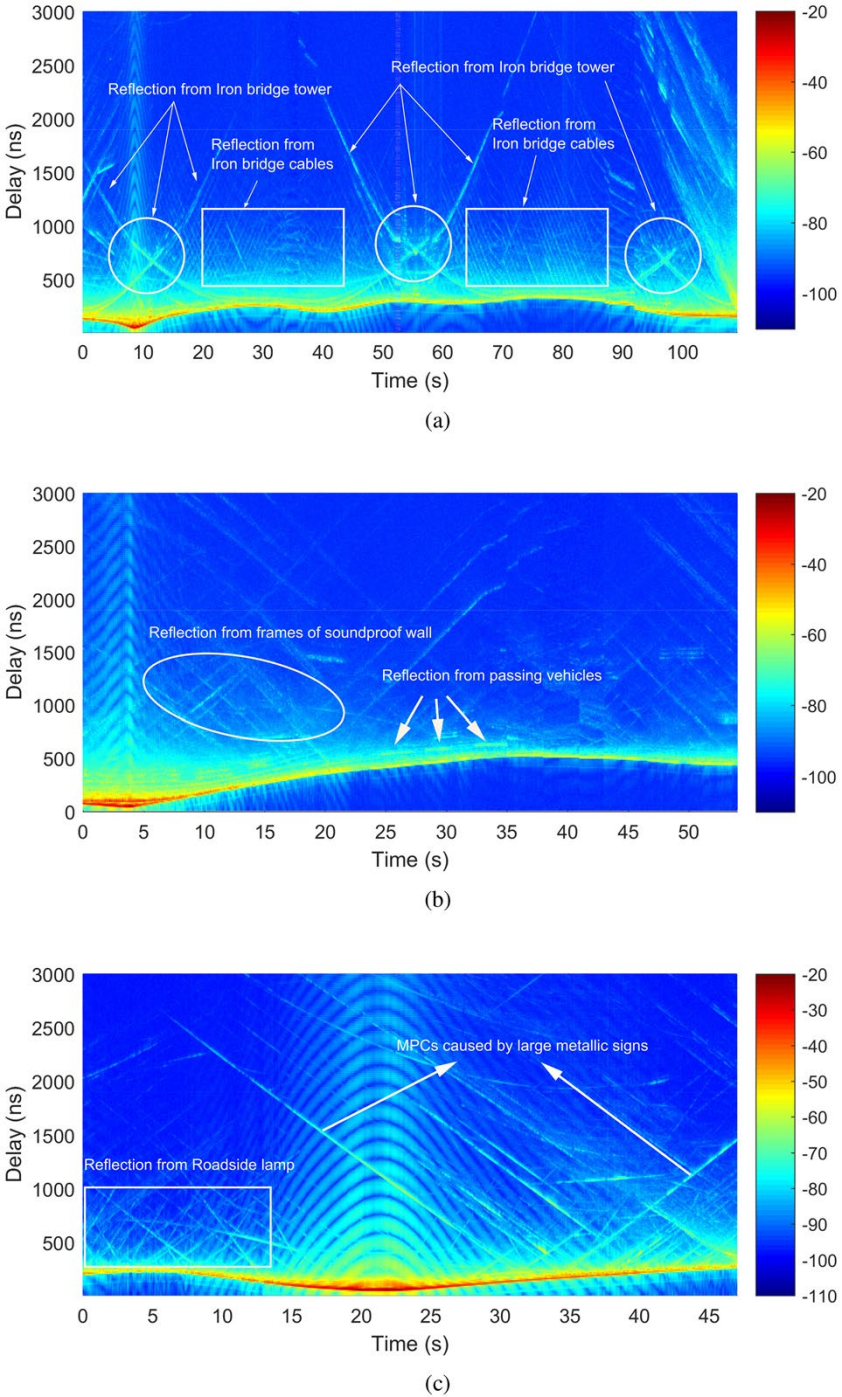
Power delay profiles of the measured scenarios. (**a**) Iron bridge. (**b**) Soundproof wall. (**c**) Roadside lamp.

Based on the wireless propagation principle and measurement results, we extract the instantaneous PDPs for the three measurement scenarios. The instantaneous PDPs over measurement time are shown in Fig. 3. The result also presents the relationship between received power and delay, from which the multi-path phenomena caused by different scatterers can be observed in the three measurement scenarios.

For the measurement 1 (iron bridge case), some MPCs with strong power and large lifetime can be observed, which are marked by white circles in Fig. 3a. These MPCs are caused by the cable towers on the iron bridge. It can also be observed that measurement vehicles approaching the cable towers will result in MPCs with small delay and strong power, and vehicles leaving the cable tower will lead to MPCs with large delay and weak power. The largest delay can reach 2500 ns with a corresponding propagation distance of 750 m. In addition, MPCs can also be caused by the iron bridge cables, however, with a short lifetime and a relatively weak power. In order to distinguish from the foregoing components, these weak MPCs are marked by the white squares in Fig. 3a.

For the measurement 2 (soundproof wall case), the main MPCs are produced by the iron frames of the soundproof walls, such as paths marked by white ellipse in Fig. 3b. The frames of soundproof walls are fixed on both sides of the road. Thus, the delay and power of MPCs change with the vehicles approaching or leaving the iron frames. Meanwhile, other passing vehicles can lead to MPCs as well. However, the MPCs originated from other vehicles have a small delay, weak power and short lifetime, as marked by the white arrows in Fig. 3b. In addition, the influence from high buildings beside the road can also be observed occasionally.

For the measurement 3 (road lamp case), the metal road lamp poles are densely distributed on both sides of the road, which result in a large number of MPCs. These propagation paths have a relatively small delay of about 1000 ns with a corresponding propagation distance of 300 m. We use white square to highlight these MPCs, as shown in Fig. 3c. Due to the measurement conducted on an open bridge over a lake, the influence from the large traffic signs can also be observed obviously. Meanwhile, it can be observed that the MPCs caused by large metallic traffic signs have a large delay and a relatively strong power, which are marked by the white arrows in the figure.

From the analysis above, it can be found that the MPCs are generally produced by surrounding scatters, especially the scatters covered by metallic surface or with large size. In the three measurements, the iron cable tower of the bridge, the large metallic traffic signs, and large buildings result in rich MPCs with large delay and strong power. Meanwhile, the iron bridge cables, metallic frames of soundproof walls, road lamp poles, and passing vehicles can also lead to MPCs. However, these MPCs have a small delay and a short lifetime due to the small size of the scatterers. But in any case, MPCs caused by both cases can make an obvious impact on the vehicular channel characteristics.

**Figure 4:**
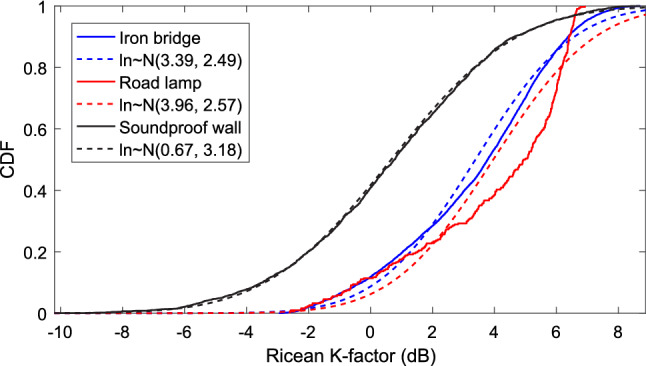
Cumulative distribution function of the Ricean K-factor.

In the analysis of wireless channel, Ricean K-factor is an important parameter to present the ratio relationship of the power between specular and diffuse components. It is widespread for characterizing the small-scale fading. The definition of Ricean K-factor can be described by equation (6).

6 $$\begin{aligned} K_{{({\mathrm{dB}})}}=10\cdot {{\mathrm{log}}}_{10}\left( \frac{r^2}{2\sigma ^2}\right) \end{aligned}$$

where, $r^2$ and $2\sigma ^2$ are the power of specular part (line of sight component) and diffuse part (other MPCs except LOS component), respectively.

According to the measurement results, the Ricean K-factor for the three measurements are extracted and the cumulative distribution functions (CDFs) of them are presented in Fig. 4. It can be found that the mean of Ricean K-factor for the 3 measurements in iron bridge, soundproof wall, and road lamp scenarios are 3.39 dB, 0.67 dB, and 3.96 dB with standard deviation of 2.49 dB, 3.18 dB, and 2.57 dB, respectively. The results indicate that average values of the Ricean K-factors in the iron bridge and road lamp scenarios are larger than that in the soundproof wall scenario. The reason is that the relative openness of propagation environments in the iron bridge and road lamp cases make the LOS component dominant. We thus get a bigger value of Ricean K-factor. On the contrary, the propagation environment in the soundproof wall case is a semi-closed one. The influence of the multipath effect from the passing vehicles is obvious. This can also be found from the large standard deviation of Ricean K-factor, which indicates that the process affected by MPCs is varying.

### RMS delay spread and RMS Doppler spread

Similar with the acquisition process of PDP, the discreted CIRs within each quasi-stationary window can be employed to acquire the delay-Doppler spectrum by equation (7).

7 $$\begin{aligned} S(r \Delta \nu ,\Delta \tau _{\min } m) = |f_{\mathrm {dft}}[h(T_{\mathrm {C}} n, \Delta \tau _{\min } m)]|^{2} \end{aligned}$$

where $\nu = r\cdot \Delta \nu$ denotes Doppler frequency shift with a unit of Hz. $f_{\mathrm {dft}}[  \cdot  ]$ expresses discrete Fourier transform (DFT). $\Delta \nu$ represents Doppler resolution. Parameter $r = \{r_{\min }, r_{\min }+1, r_{\min }+2,\ldots ,r_{\max }\}$ is a scope value.

Similarly, the average delay-Doppler spectrum $P_B(t_j,r \Delta \nu )$ can be obtained on the basis of equation (7), according to the same operation method between equation (4) and (5).

In the analysis of wireless channel, the appearance of delay and frequency dispersion is related to root means square (RMS) delay spread and RMS Doppler spread. So these are two important channel characteristics. Generally, they can be defined as the second central moments of PDP and delay-Doppler spectrum, respectively, shown in equation (8) and equation (10).

8 $$\begin{aligned} S_{\tau }(t_{j}) = \sqrt{\frac{\sum \limits _{m=1}^{M_{\mathrm {D}}}(\Delta \tau _{\min } m)^2 \cdot {\overline{P}}(t_{j},\Delta \tau _{\min } m)}{\sum \limits _{m=1}^{M_{\mathrm {D}}}{\overline{P}}(t_{j},\Delta \tau _{\min }m)}-[T_{\mathrm {m}}(t_{j})]^2} \end{aligned}$$

where $T_{\mathrm {m}}(t_{j})$ denotes the mean delay of the *j*-th window, as

9 $$\begin{aligned} {T_{\mathrm {m}}(t_{j}) = \frac{\sum \limits _{m=1}^{M_{\mathrm {D}}}(\Delta \tau _{\min }m) \cdot {\overline{P}}(t_{j},\Delta \tau _{\min } m)}{\sum \limits _{m=1}^{M_{\mathrm {D}}}{\overline{P}}(t_{j},\Delta \tau _{\min } m)}} \end{aligned}$$

The RMS Doppler spread of the *j*-th window $S_{\nu }(t_{j})$ can be obtained by equation (10)

10 $$\begin{aligned} S_{\nu }(t_{j}) = \sqrt{\frac{\sum \limits _{r_{\min }}^{r_{\max }}(r \Delta \nu )^2 P_{\mathrm {B}}(t_{j}, r \Delta \nu )}{\sum \limits _{r_{\min }}^{r_{\max }}P_{\mathrm {B}}(t_{j}, r \Delta \nu )}-[\nu _{\mathrm {m}}(t_{j})]^2} \end{aligned}$$

where $\nu _{\mathrm {m}}(t_{j})$ expresses mean Doppler frequency shift of the *j*-th window, as

11 $$\begin{aligned} \nu _{\mathrm {m}}(t_{j}) = \frac{\sum \limits _{r_{\min }}^{r_{\max }}(r \Delta \nu ) \cdot P_{\mathrm {B}}(t_{j}, r \Delta \nu )}{\sum \limits _{r_{\min }}^{r_{\max }}P_{\mathrm {B}}(t_{j}, r \Delta \nu )}. \end{aligned}$$

**Figure 5:**
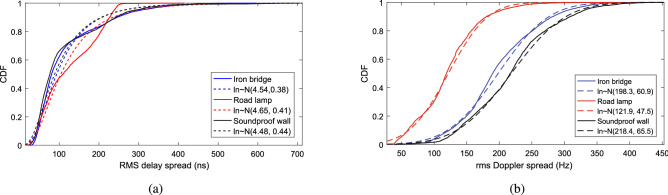
Cumulative distribution function of the rms delay spread and the rms Doppler spread. (**a**) rms delay spread. (**b**) rms Doppler spread.

In addition, in the analysis of RMS delay and RMS Doppler spreads, a threshold should be set to avoid influence from spurious components. According to the reference by $Fang\,et.\, al$^20^, we set the threshold to 6 dB above the average noise floor in our analysis. Then, based on the measurement results, the CDFs of RMS delay spread and RMS Doppler spread of the three measurements are presented in Fig. 5a,b. The statistical results show that the average values of RMS delay spread in iron bridge, soundproof wall, and road lamp cases are 4.54 ns, 4.48 ns, and 4.65 ns with a standard deviation of 0.38 ns, 0.44 ns, and 0.41 ns, respectively. It can be observed that the difference of influence from the surroundings on the RMS delay spread is not very obvious in our 3 measurements.

From Fig. 5b, it can be found that the largest average RMS Doppler spread of 218.4 Hz with a largest standard deviation of 65.5 Hz appears in the measurement of the soundproof wall scenario. This is consistent with the finding in the analysis of Ricean K-factor, which is caused by the semi-closed propagation environment and the influence of multi-path effect from the passing vehicles.

## Cluster identification and statistical results

According to the principle in reference by *Molisch*^21^, MPCs often tend to arrive at the receiver in the form of ”cluster”. Therefore, a classical Saleh-Valenzuela (SV) model^22^ was proposed to characterize the relationship between received power and delay of clusters.

12 $$\begin{aligned} \begin{aligned} PDP(\tau )&=\sum \limits _{l=1}^{L}\sum \limits _{k=1}^{K_{l}} \Bigg \{\bigg [20\log _{10}(c_{1,1})-\left( \frac{T_{l}}{\Gamma } +\frac{\tau _{k, l}}{\gamma }\right) \\&\quad \cdot 10\log _{10}(\mathrm {e})\bigg ]\cdot \delta \left( \tau -T_{l}-\tau _{k, l}\right) \Bigg \}\\ \end{aligned} \end{aligned}$$

where $c_{1,1}$ represents the amplitude of the 1-st MPC in the 1-st cluster. *L* and $K_{l}$ denote the total number of clusters in the instantaneous PDP and total number of MPCs in the *l*-th cluster, respectively. $T_{l}$ is the arrival delay of the 1-st path in the cluster *l*. For the *l*-th cluster, $\tau _{k, l}$ represents the excess delay of the *k*-th path relative to the 1-st path by definition of $\tau _{1, l}=0$. $\Gamma$ and $\gamma$ represent the cluster decay time constant and the intracluster decay time constant, respectively.

**Figure 6:**
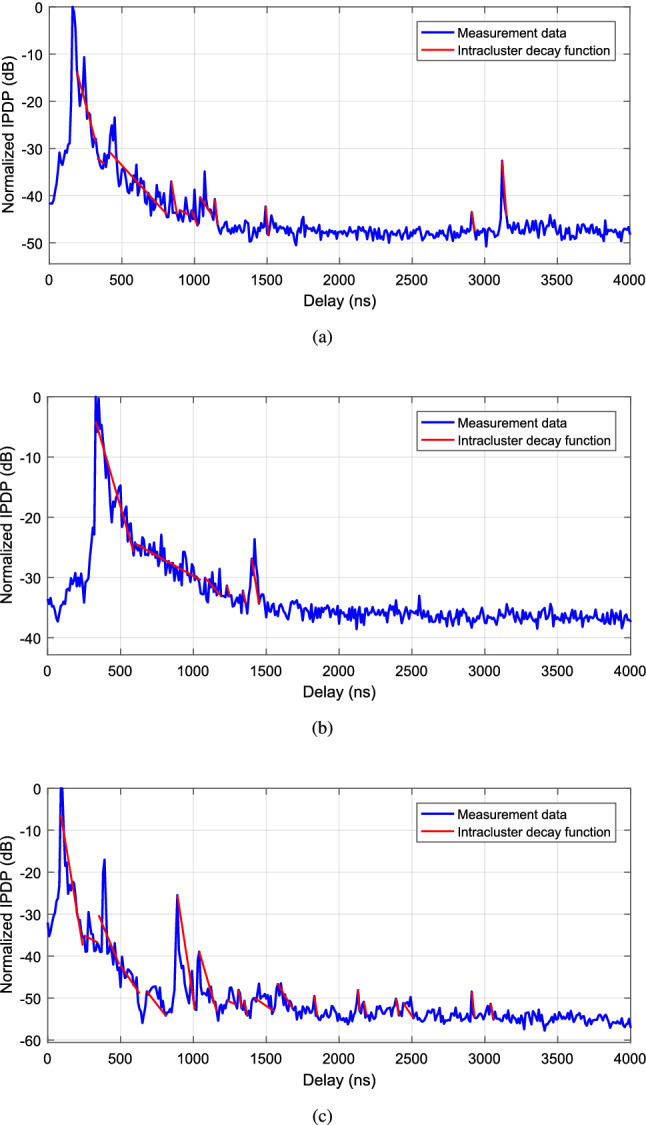
Result of cluster identification for the measurements. (**a**) Iron bridge. (**b**) Soundproof wall. (**c**) Roadside lamp.

Generally, the purpose of PDP modeling can be considered to obtain the arrival delay of the 1-st path in the *l*-th cluster $T_{l}$, the cluster decay time constant $\Gamma$ and the intracluster decay time constant $\gamma$, i.e., the identification of clusters. Therefore, we adopt the methodology based on the theory of Kurtosis and region competition in reference by *Gentile*^23^ to realize the cluster identification. Results of the cluster identification for the three measurements are presented in Fig. 6. It is observed that the LOS and MPC clusters can be identified well. Meanwhile, we can also find that the delay distributions of most MPC clusters are concentrated within or around 1000 ns, which means the main influence of MPCs is from scatters around 300 m for vehicular communications. However, there is still difference between the distribution of the MPCs due to the different surroundings in the three measurement scenarios. In the iron bridge case, we can observe that some MPCs have non-ignorable energy with a large delay, such as the reflection path with a delay of 3120 ns with a power of $-32.6 \,\hbox {dB}$ (the corresponding propagation distance is 936 m). And in the road lamp case, we can observe some reflection paths with a large delay, but the propagation energy is relatively weak (such as the paths at 2130 ns with a power of $-48.1 \,\hbox {dB}$ and at 2910 ns with a power of $-48.46 \,\hbox {dB}$). Compared with these two cases, there is rare reflection path with a large delay in the soundproof wall scenario, which only one path at 1420 ns with a power of $-23.6 \,\hbox {dB}$ can be observed. The reason is that the propagation environment is relatively closed due to the existence of soundproof walls on both sides of the road.

In the cluster identification and modeling of PDP, the cluster decay time constant and the intracluster decay time constant are two important parameters. In this paper, we also make analysis on the two key parameters by extracting inter-cluster interval and reciprocal of intracluster decay time constant. The inter-cluster interval between the (*l*-1)-th cluster and the *l*-th cluster is defined as $\Delta T_l = T_l-T_{l-1} (l\ge 2)$. And reciprocal of intracluster decay time constant is an important part of the ray decay function.

**Figure 7:**
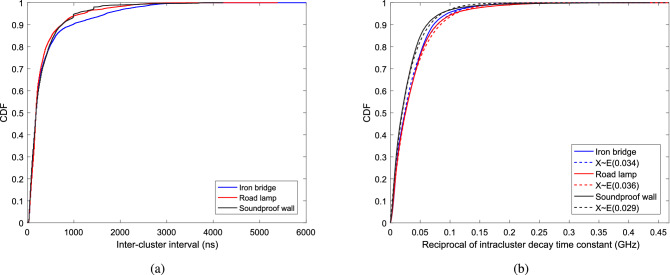
Statistical results of the inter-cluster interval and the intracluster decay constant. (**a**) Inter-cluster interval. (**b**) Reciprocal of intracluster decay time constant.

Fig. 7 presents the statistical results of the inter-cluster interval and the intracluster decay constant. It can be found that there is a small difference in inter-cluster intervals between the results obtained from the measurements of soundproof wall and road lamp cases. 90% of inter-cluster interval is within 740 ns for both the results extracted from them. However, 90% of inter-cluster interval is within 970 ns in the measurement of iron bridge scenario. This is caused by the reflection from the iron tower with a large delay and a strong power. The same finding can be observed from the statistical results of reciprocal of intracluster decay time constants. In addition, the result indicates that the reciprocal of intracluster decay time constants follow the exponential distribution with rate parameters of 0.034 GHz, 0.029 GHz, and 0.036 GHz, respectively.

## Conclusion

This paper discusses the non-stationary characteristics of vehicular channel for different roadside scattering environments, focusing on the influence from different scatterers. The measurement data are collected from three V2V channel measurements in different scattering environments, including iron bridge, soundproof wall, and road lamp scenarios. The stationary time and frequency, power delay profile, Ricean K-factor, RMS delay spread and RMS Doppler spread are extracted. Considering the birth-death process of MPCs, cluster identification is also conducted. The analysis results of the stationary time and frequency show that the V2V channel in soundproof wall scenario is more stable than that in iron bridge and road lamp scenarios. From the analysis results of MPCs, it can be found that the metallic cable tower on the bridge can cause rich MPCs with large delay and strong power. The metallic frames of soundproof walls and the poles of road lamps can lead to MPCs as well, however, with small delay and weak power due to their small sizes. Meanwhile, in the relatively closed propagation environment of the soundproof wall case, the influence from other passing vehicles cannot be ignored. In addition, it also can be found that a large metallic cable tower can make a large inter-cluster interval and reciprocal of intracluster decay time constant. These results can provide reference for the vehicular wireless communication network design and optimization.

## Data Availability

The datasets used and/or analysed during the current study available from the corresponding author on reasonable request.
